# Supplementary material for: A Straightforward Cytometry-Based Protocol for the Comprehensive Analysis of the Inflammatory Valve Infiltrate in Aortic Stenosis
Source: Int J Mol Sci. 2023 Jan 22;24(3):2194. doi: 10.3390/ijms24032194 (PMC9916774; doi:10.3390/ijms24032194)
Supplement: Supplementary file 1 [file ijms-24-02194-s001.zip › ijms-2160619-supplementary.pdf]

Table S1. Infiltrating valve cells phenotyping panel

ANTIBODY	CLON	FLUOROCHROME	SUPPLIER
CD45	HI30	Alexa Fluor 700	BD Becton Dickinson
CD3	SK7	PE-Cy5.5	Invitrogen
CD8	RPA-T8	V500	BD Becton Dickinson
CD4	SK3	BB700/BV510	BD Becton Dickinson
CD14	TÜK4	APC	BD Becton Dickinson
CD28	CD28.2	PE-Cy7	BD Becton Dickinson
CD16	3G8	BV786	BD Becton Dickinson
CD56	NCAM16.2	BV421	BD Becton Dickinson
Live/Dead Fixable Near-IR Dead Cell Stain Kit	NA	Near-IR	Molecular Probes of Life Technologies

Table S2. Infiltrating valve cells phenotyping panel for functional assay

	ANTIBODY	CLON	FLUOROCHROME	SUPPLIER
Surface	CD45	HI30	Alexa Fluor 700	BD Becton Dickinson
	Live/Dead Fixable Near-IR Dead Cell Stain Kit	NA	Near-IR	Molecular Probes of Life Technologies
	CD8	RPA-T8	V500	BD Becton Dickinson
	CD4	SK3	BUV395	BD Becton Dickinson
ICS	IFN γ	45-15	FITC	Miltenyi
	Granzyme B	GB11	PE	BD Becton Dickinson
	TNF	MAb11	BV650	BD Becton Dickinson
	CD3	SK7	PE-Cy5.5	Invitrogen

Table S3. Peripheral T blood cells phenotyping panel

	ANTIBODY	CLON	FLUOROCHROME	SUPPLIER
Surface	CD45	HI30	Alexa Fluor 700	BD Becton Dickinson
	CD3	SK7	PE-Cy5.5	Invitrogen
	CD8	RPA-T8	APC-H7	BD Becton Dickinson
	CD4	SK3	BB700	BD Becton Dickinson
	CD56	NCAM16.2	BV421	BD Becton Dickinson
	CD28	CD28.2	PE-Cy7	BD Becton Dickinson

Table S4. Peripheral innate blood cells phenotyping panel

	ANTIBODY	CLON	FLUOROCHROME	SUPPLIER
Surface	CD45	HI30	Alexa Fluor 700	BD Becton Dickinson
	CD3	SK7	PE-Cy5.5	Invitrogen
	CD56	NCAM16.2	BV421	BD Becton Dickinson
	CD19	SJ25C1	BV650	BD Becton Dickinson
	CD7	M-T701	APC	BD Becton Dickinson
	CD16	3G8	BV786	BD Becton Dickinson
	CD14	TÜK4	APC Vio770	BD Becton Dickinson

Table S5. Mean and standard deviation of frequencies of innate and adaptative leukocyte subpopulations and phenotypes.

			Valve	PB
			Mean ± SD	
**Neutrophils CD16+**			7,76 ± 7,11	57,84 ± 17,87
**Monocytes**			16,03 ± 6,83	4,83 ± 3,68
**NK cells**			5,18 ± 3,06	3,54 ± 2,65
	**CD56bright**		29,75 ± 15,57	7,38 ± 5,44
	**CD56dim**		15,06 ± 10,52	83,46 ± 9,13
	**CD56–CD16+**		4,99 ± 5,84	5,93 ± 5,91
**T cells**			48,09 ± 10,26	20,47 ± 10,22
	**CD4**		56,38 ± 10,39	61,23 ± 14,77
		**CD56**	7,65 ± 7,51	2,53 ± 4,51
		**CD28null**	27,73 ± 14,45	11,77 ± 16,63
		**CD28**	82,27 ± 14,45	88,23 ± 16,63
	**CD8**		39,21 ± 10,01	34,24 ± 14,77
		**CD56**	14,88 ± 9,11	22,59 ± 14,61
		**CD28null**	59,74 ± 14,94	58,66 ± 22,57
		**CD28**	41,26 ± 14,94	41,34 ± 22,57
	**DN**		2,86 ± 1,75	3,99 ± 3,62
		**CD56**	17,53 ± 9,95	40,74 ± 18,51
		**CD28null**	61,46 ± 17,58	65,54 ± 21,25
		**CD28**	39,54 ± 17,58	34,46 ± 21,25
	**DP**		1,07 ± 1,7	0,54 ± 0,43

*Frequencies of neutrophils, monocytes, NK cells, and T cells were calculated from the total of leukocytes. Frequencies of NK and T cell subsets and their phenotypical markers were calculated from the parent population.

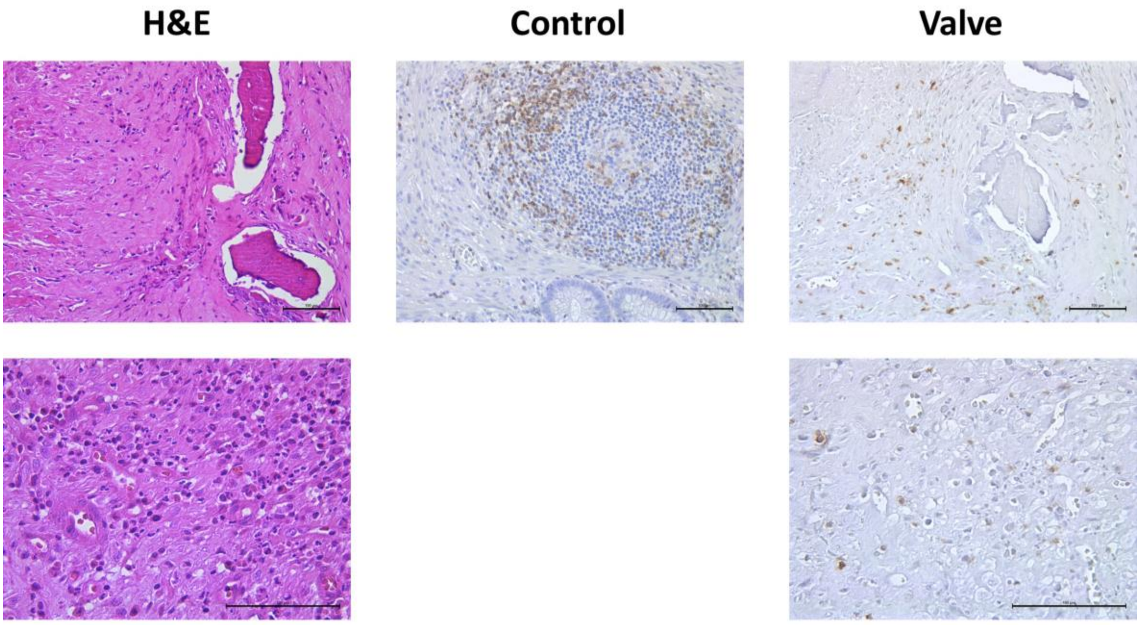

Figure S1. Immunohistochemical analysis of aortic stenosis valve sections. Aortic valve sections were stained using haematoxylin-eosin (H&E) and anti-CD3 (Valve). CD3+ cells were represented with yellow colour. Human lymph node stained with anti-CD3 was used as positive control.

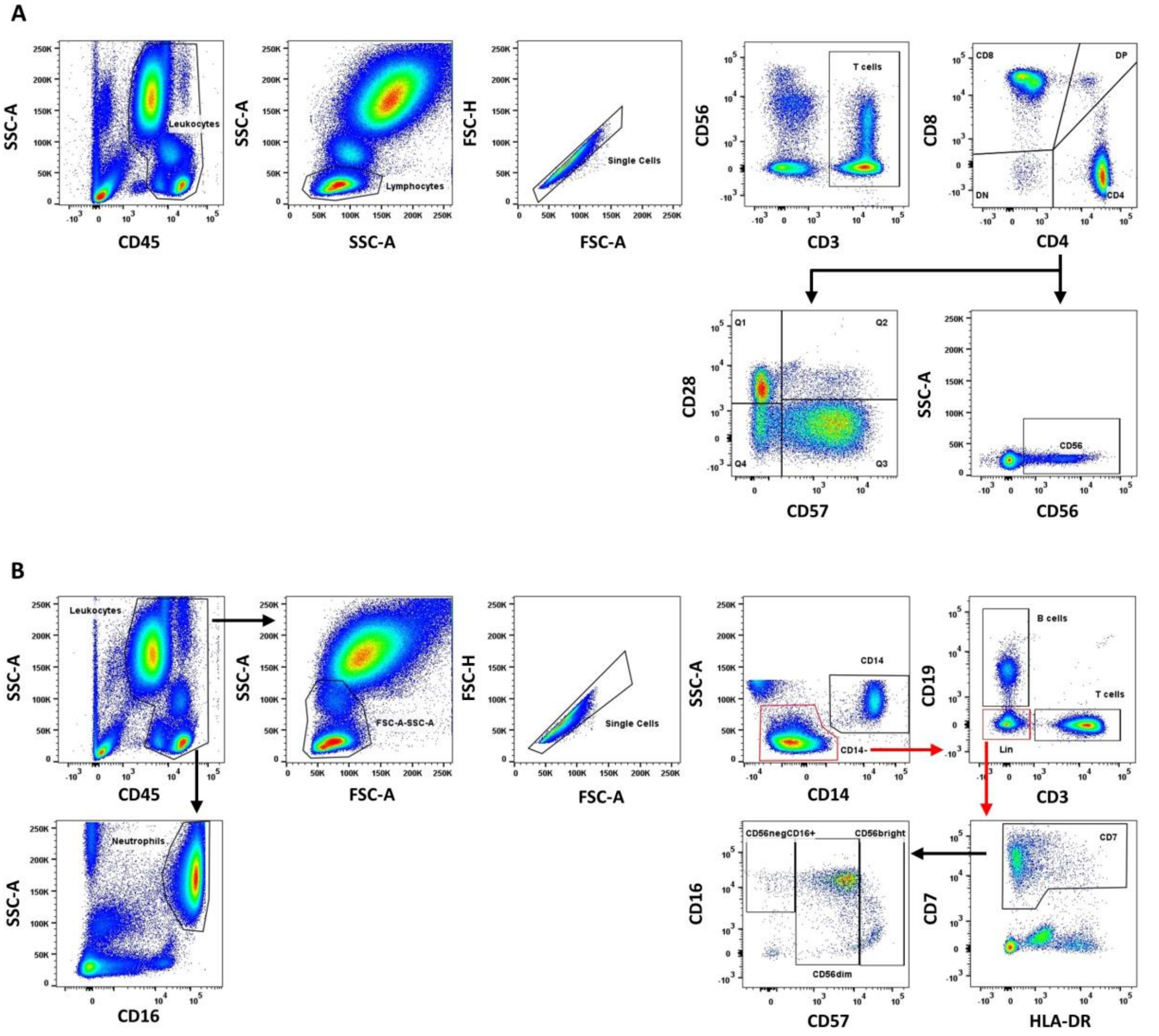

Figure S2. Gating strategy for the characterization of peripheral blood immune cells. The leukocyte gate was created in an SSC-A/CD45 plot. **(A)** Gating strategy for T blood cell characterisation. After the Leukocytes gate, lymphocytes were gated in an SSC-A/FSC-A plot. Doublets were excluded using an FSC-A/FSC-H plot. From this gate, T cells were gated confronting CD56 and CD3 markers. T cell subsets were defined in a CD8/CD4 plot. From each T cell subpopulation, CD56 was single-gated, and CD28 and CD57 markers were determined using a quad. **(B)** Gating strategy for innate blood cell characterisation. From the Leukocytes gate, the neutrophils gate was created in an SSC-A/CD16 plot. For the rest of the innate populations, monocytes and lymphocytes region were gated in an SSC-A/FSC-A plot. Doublets were excluded using an FSC-A/FSC-H plot. Monocytes (CD14) were gated in an SSC-A/CD14 plot. From cells without CD14 expression (CD14-), we excluded T and B cells in a CD3/CD19 plot. Then, we gated NK cells in a CD7/HLA-DR plot. Finally, NK cell phenotype was determined in a CD56/CD16 plot by CD56 fluorescence intensity and expression of the CD16 marker.

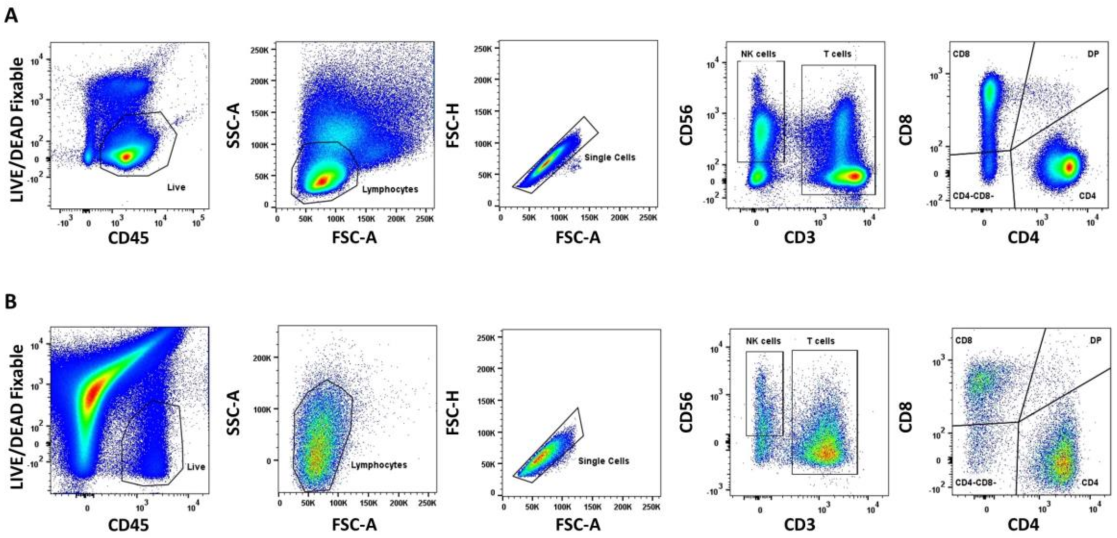

Figure S3. Gating strategy for functional assay. (A) Gating strategy for peripheral blood mononuclear cells (PBMCs) functional assay. **(B)** Gating strategy for valvular infiltrating cells. Living leukocyte gate was created in a Live-Dead Fixable/CD45 plot. Lymphocytes gate was created in an SSC-A/FSC-A plot. Then, doublets were excluded in an FSC-H/FSC-A plot. From the singlets gate, T cells and NK cells were gated in a CD56/CD3 plot. Finally, T cell subsets were determined in a CD8/CD4 plot using a quad.
